# Isolated Fusobacterium nucleatum Growth in the Blood Culture of a Middle-Aged Man With Lumbar Discitis, Surrounding Psoas Abscesses, and an Inferior Vena Cava Thrombus

**DOI:** 10.7759/cureus.55306

**Published:** 2024-03-01

**Authors:** Lucy Bomphrey, Amber Hayden, Aiden J Plant

**Affiliations:** 1 Medicine, Walsall Manor Hospital, Walsall, GBR; 2 Microbiology, Black Country Pathology Services, Wolverhampton, GBR

**Keywords:** abscess, thrombus, fusobacterium, osteomyelitis, discitis

## Abstract

Pyogenic spinal infections (PSI) have an incidence of 0.5-2.2 cases per 100,000 population, though diagnosis can be delayed by up to three months. The incidence of *Fusobacterium nucleatum* bacteremia is rare, occurring in 0.22-0.34 cases per 100,000 population, whilst its implication in spinal infections is rarer still. A man in his 60s with a background of chronic lower back pain presented to the emergency department with a two-week history of worsening back pain associated with fever and difficulty voiding. He was initially managed as pyelonephritis due to the recent history of urinary tract infection (UTI) with fever and flank pain. However, there were radiculopathy and bilateral pain on hip flexion with reduced power on the right side. The light-touch sensation was reduced over the right hallux and distal L4 dermatome. These neurological deficits associated with deranged infective markers made a diagnosis of discitis plausible. Discitis and native vertebral osteomyelitis (NVO) should be suspected in patients reporting a fever and back pain of recent onset or increasing in severity. Once discitis was confirmed, the patient was subsequently tested for tuberculosis (TB) using a T-SPOT, human immunodeficiency virus (HIV), hepatitis B virus, and hepatitis C virus, with no positive findings, but in the days following, blood cultures yielded *F. nucleatum*. Guided by knowing the natural reservoirs in the body, establishing the source of *F. nucleatum* could be achieved through head and neck imaging and investigating the gastrointestinal tract for malignant or inflammatory processes.

## Introduction

Pyogenic spinal infection (PSI) encompasses a broad spectrum of conditions including discitis, vertebral osteomyelitis, and epidural abscesses. PSI has an incidence of 0.5-2.2 cases per 100,000 population, though diagnosis can be delayed by up to three months due to its non-specific presentation [1]. Patients typically present with fever and back pain which may be mistaken to have a degenerative aetiology. The incidence of *Fusobacterium nucleatum* bacteremia is rare, occurring in 0.22-0.34 cases per 100,000 population, whilst its implication in spinal infections is rarer still [2,3]. *F. nucleatum* is an obligate anaerobic Gram-negative bacillus with reservoirs in the gastrointestinal tract and oral cavity [4]. Of the published cases identifying spinal infections attributable exclusively to* F. nucleatum*, most are associated with abscesses, and one case reported concurrent thrombosis. Herein, we describe the rare involvement of* F. nucleatum* in complex discitis and native vertebral osteomyelitis (NVO) with large vessel thrombus and suggest investigations for identifying an infection source based on the current literature.

## Case presentation

A retired man in his 60s with a background of chronic lower back pain, depression, and myocardial infarction presented to the emergency department with a two-week history of worsening back pain associated with fever and difficulty voiding. He reported a recent urinary tract infection (UTI) for which he had received three days of antibiotics thus far. The patient experienced pain in the right lumbar region which radiated in a band-like distribution to the groin, encircling the pelvis. The dysesthesia was described as electric shock-like and restricted movement but was also profoundly worse when recumbent. 

During systematic questioning, the patient reported multiple red flag symptoms alluding to a more sinister underlying pathology. He had experienced a two-week history of night sweats, 10 kg of unintentional weight loss, and general malaise over one month. His difficulty in voiding was accompanied by a change in bowel habits, including two weeks of loose stools, vomiting, and one episode of faecal incontinence. Neurological questioning revealed a recent gait abnormality necessitating a walking stick, sensation of leg weakness, and loss of balance, with paraesthesia in the feet and toes. He was now unable to climb the stairs in his home.

On examination, he was tender in the right flank and suprapubic region. Hip flexion elicited lumbar pain bilaterally with reduced power on the right side. The light-touch sensation was reduced over the right hallux and distal L4 dermatome. Examination of the spine identified warmth and tenderness over the right lumbosacral joint. The rectal examination revealed an inability to bear down, though the sensation was intact. Blood results showed a raised white blood cell count of 20.8 (10^9^/L), neutrophils 17.7 (10^9^/L), monocytes 1.7 (10^9^/L), and C-reactive protein 228 (mg/L). Lymphocytes were normal at 1.2 (10^9^/L) as were platelets at 216 (10^9^/L) showing an infective picture. 

He was initially managed as pyelonephritis due to the recent history of UTI with fever and flank pain. However, the presence of radiculopathy and mild neurological deficit associated with deranged infective markers made a diagnosis of discitis plausible. Furthermore, the long-term systemic symptoms raised suspicion of gastrointestinal tract malignancy. Further investigations included blood and urine cultures in addition to computed tomography (CT) of the thorax, abdomen, and pelvis to exclude malignancy.

**Figure 1:**
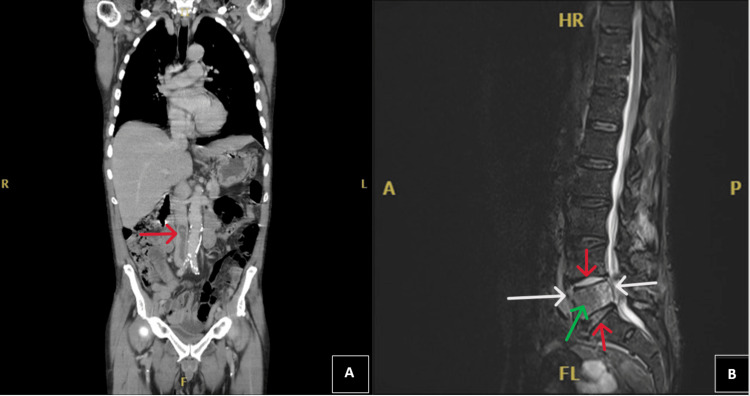
CT and MRI of the patient (A) CT of the thorax, abdomen, and pelvis. Coronal view showing an air-containing thrombus extending from the left common iliac vein to the IVC, marked with a red arrow. (B) MRI of the whole spine. Sagittal view showing emphysematous osteomyelitis of the L5 vertebral body (green arrow) and L4/L5 and L5/S1 discitis (red arrows). A prevertebral collection and epidural collection (white arrow) within the spinal canal are also seen at the same level CT: computed tomography; MRI: magnetic resonance imaging; IVC: inferior vena cava

The CT revealed an air-containing thrombus extending from the left common iliac vein to the inferior vena cava (IVC) (Figure 1A) and vertebral body abnormalities in L5. There was a small cyst in the right lobe of the liver, but no other findings. Magnetic resonance imaging (MRI) of the whole spine was recommended to further evaluate for discitis which identified multiple abnormalities in addition to the IVC thrombus, including L5 bone marrow oedema with L4/L5 and L5/S1 discitis. An adjacent epidural collection within the spinal canal is seen, causing mild thecal sac and nerve root compression. In addition, images showed a prevertebral and right psoas collection at the L5 level (Figure 1B).

He was subsequently tested for tuberculosis (TB) using a T-SPOT, human immunodeficiency virus (HIV), hepatitis B virus, and hepatitis C virus, with no positive findings. However, a blood culture, taken at the point of his admission to the emergency department, yielded *F. nucleatum* after 67 hours of incubation in the anaerobic bottle. Incubation under anaerobic conditions for 72 hours was required until sufficient growth allowed identification as *F. nucleatum* via matrix-assisted laser desorption ionization time-of-flight (MALDI-TOF) analysis. Antimicrobial susceptibility was determined with broth microdilution with the following minimum inhibitory concentrations (MICs): penicillin, 64 mg/L; co-amoxiclav, 0.125 mg/L; and metronidazole, <0.125 mg/L. Repeat blood cultures taken five days after admission remained sterile. Coagulation screens for pre-existing thrombophilia were negative, and transoesophageal echocardiogram (TOE) excluded infective endocarditis. A routine dental examination failed to reveal any caries requiring intervention from a dental surgeon, and there was no further head or neck imaging undertaken. 

Initial management of the patient included anticoagulation to manage the thrombus. Considering the finding of *F. nucleatum* in the blood, empirical treatment was rationalised to intravenous ceftriaxone, 2 g q12h, with oral metronidazole, 400 mg q8h. After four weeks in the hospital, his antibiotic therapy was switched to oral co-amoxiclav (1000 mg amoxicillin with 125 mg clavulanic acid, q8h), for three months in total, to facilitate discharge. At the multidisciplinary outpatient follow-up, his symptoms had resolved; interval MRI confirmed resolving NVO and IVC thrombus, and his inflammatory markers normalised. The patient's abscesses were not drained.

## Discussion

Few published cases describe the finding of *F*. *nucleatum* with discitis and NVO. A PubMed search for (*F.* *nucleatum*) AND (discitis OR vertebral osteomyelitis OR spinal infection) yielded 27 results since the 1970s. Of the 27 identified publications, the authors found 15 published reports of discitis or NVO attributable exclusively to *F*. *nucleatum* [5-8]. To our knowledge, this is the first with an IVC thrombus. 

The lumbar spine was the most common location of spinal infections related to *F*. *nucleatum*, and most cases were associated with abscesses. Whilst some patients were immunocompromised, many were not. A significant proportion of *F*. *nucleatum*-associated NVO cases suggest origins of infection arising from the oropharyngeal, respiratory, or dental routes [6-9]. Dissemination of *F*. *nucleatum* appears to be due to its ability to bind with and invade host cells via a specific adhesin FadA, which is regarded as one of its virulence factors [4,10]. FadA binds to host cell cadherins, present in endothelial and epithelial tissue, and enables *F*. *nucleatum* to disseminate [4,10]. Once in the bloodstream, *F*. *nucleatum* then can induce inflammation via pro-inflammatory cytokines [4,10]. The combination of pro-inflammatory properties with endothelial disruption contributes to its thrombotic tendency observed in other cases of *F. nucleatum *bacteremia [7]. The subsequent clotting dysregulation from *F*. *nucleatum* bacteremia is suggested as the cause for this patient's IVC and left common iliac thrombus, in the absence of pre-existing clotting disorders [7]. After searching the literature, this is the second case published of isolated* F. nucleatum* discitis with associated thrombosis [7].

Large quantities of *F*. *nucleatum* in stool samples have been associated with colorectal cancer, and there is evidence to suggest that it has a key role in inflammatory and malignant processes, as both a passenger and a driver in tumour invasion [4,10]. *F*. *nucleatum* is also known to cause periodontitis; therefore, another possible aetiology is seeding to the lumbar region from the oral cavity [6]. There were no concerns about our patient's oral hygiene so this was deemed a less likely infection source. To identify a focus or route for the invasion of *F. nucleatum*, colonoscopy or head and neck imaging could be necessary [5,7,9,11]. Specifically, CT of the head with contrast study is an optimal choice to investigate for oropharyngeal infections [12]. We also suggest taking a thorough dental history and examination [6,9].

An underlying reason for *F*. *nucleatum* bacteremia has not been confirmed in this patient; however, the authors hypothesise that due to the change in bowel habit, two weeks of loose stools, and vomiting, this patient developed NVO due to bacteremia arising from the gastrointestinal tract. This likely caused haematogenous and contiguous spread, allowing the organism to cause multiple abscesses around the L4/L5 spinal level and infect nearby structures, including the intervertebral discs and vertebral bodies.

The diagnosis of NVO itself is frequently delayed, with the pain mistaken to have a degenerative aetiology [13]. NVO should be suspected in patients reporting a fever and back pain of recent onset or increasing in severity [13]. Pain may radiate into the abdomen or groin, and a key characteristic raising suspicion of NVO is radiculopathy, as it did in this case. Owing to clinical urgency and this patient presenting out of hours, CT was deemed most clinically appropriate to assess for pathology; however, MRI is the modality of choice due to its high sensitivity and specificity. That said, CT can quantify the severity of bone and soft tissue involvement and raise suspicion of NVO in the first instance of this case [13]. Once the spinal infection has been confirmed, enquiring into the patient's social history addresses the presence of risk factors for immunocompromised or vulnerable states that can facilitate spinal infection [13]. *Staphylococcus aureus* contributes to the majority of NVO and discitis being present in almost 50% of the cases [13], but direct questioning about travel, hobbies, animal contact, and ingestion of unpasteurized milk can implicate specific causative agents such as melioidosis, brucellosis, salmonellosis, Q fever, and mycobacteria. 

In severe NVO cases, indications for surgery include neurological deficits or symptoms of cord compression [13]. Whilst our patient did have neurological changes, supported by thecal sac compression seen on axial imaging, surgical intervention was not undertaken, given the deficits were not progressive and repeat imaging did not reveal instability or deformity in the vertebral body [13]. Furthermore, access to L4/L5 is made markedly more challenging by the pelvic brim. This rationale also justifies managing the psoas abscess conservatively. 

## Conclusions

Discitis and NVO should be considered in patients with new or worsening back pain and fever, to prevent delays in treatment. The bacterium grown in this case is rare, and its ability to cause spinal infections is associated with gastrointestinal and periodontal pathologies, as well as immunosuppression. This mandates further testing and specialist follow-up to identify any risk factors for *F*. *nucleatum* bacteremia. Guided by knowing the natural reservoirs in the body, we can suggest that establishing the source of *F.* *nucleatum* in patients with an unknown infection source could be achieved through head and neck imaging and investigating the gastrointestinal tract for malignant or inflammatory processes via colonoscopy. There appear to be increasingly recognized sequelae of disease features associated with *F*. *nucleatum*, including thromboembolisms and abscesses.
